# Enhancing Healing Strategies: Negative-Pressure Wound Therapy and Delayed Primary Closure in Abdominal Wounds Post-emergency Laparotomy for Intestinal Perforation

**DOI:** 10.7759/cureus.60738

**Published:** 2024-05-21

**Authors:** Vivek Belsariya, Meghraj Kundan, Vekhotso Nyekha, Nemi Chandra J

**Affiliations:** 1 General Surgery, Vardhman Mahavir Medical College and Safdarjung Hospital, New Delhi, IND

**Keywords:** negative-pressure wound therapy, abdominal laparotomy wounds, intestinal perforation, post-emergency laparotomy, delayed primary closure

## Abstract

Background

Intestinal perforation is a life-threatening condition requiring immediate surgical intervention. Surgical-site infections (SSIs) and wound dehiscence are common complications associated with emergency laparotomy for intestinal perforation. Finding optimal wound management and postoperative strategies can significantly impact patient outcomes and reduce the risk of complications. Negative-pressure wound therapy (NPWT) is a relatively recent tool employed in the care of wounds to control SSIs and foster healing.

Methodology

A prospective, observational, cohort study was conducted among 150 patients who underwent emergency exploratory laparotomy due to intestinal perforation at the general surgery department of a tertiary care hospital in New Delhi between July 2022 and December 2023. Preoperatively, all patients underwent initial resuscitation. Intraoperatively, the extent of peritonitis was determined and was categorized according to the Centers for Disease Control and Prevention (CDC) classification. Postoperatively, NPWT dressing was applied to the patient’s midline laparotomy wound on postoperative day (POD) two. Negative pressure was set at 75-125 mmHg with suction. The number of NPWT dressing changes required was documented. The wound was closed with vertical mattress sutures under local anesthesia, delayed primary closure (DPC). The incidence of SSIs, the duration for DPC, the incidence of fascial dehiscence, the number of NPWT dressing changes, and the length of hospital stay were documented according to CDC groups.

Results

The mean age in CDC categories 2, 3, and 4 were 31.789, 28.733, and 42.676 years, respectively. The most common cause of perforation was enteric fever (n = 42, 28%), followed by tuberculosis (n = 36, 24%). Most patients had no known comorbidities (n = 80, 53.3%). Overall, 16% of patients (n = 24) were both alcoholics and smokers. The most frequent bacteria in all CDC categories was *Escherichia coli*. Fourteen patients developed burst abdomen in the postoperative period and were excluded from the study. The mean duration of DPC increased with higher CDC categories, with CDC category 4 displaying the most extended mean duration at 10.70 days. The number of NPWT dressing changes increases with higher CDC categories, with CDC category 4 exhibiting the highest mean at 2.00 changes. The mean hospital stay increased with higher CDC categories, with CDC category 4 showing the most extended mean stay at 17.324 days. Statistical analysis revealed no significant association between SSI occurrence and CDC categories.

Conclusions

NPWT followed by DPC is a promising approach to managing gastrointestinal perforations, reducing SSIs, and potentially improving patient outcomes. However, further research is needed to explore the specific benefits of NPWT in conjunction with DPC and its efficacy in various clinical scenarios.

## Introduction

Intestinal perforation is a life-threatening condition requiring immediate surgical intervention. Complications such as peritonitis and sepsis can lead to high morbidity and mortality rates if not managed promptly and effectively. Finding optimal wound management and postoperative strategies can significantly impact patient outcomes and reduce the risk of complications [1]. Surgical-site infections (SSIs) and wound dehiscence are common complications associated with emergency laparotomy for intestinal perforation. These complications can prolong hospital stays, increase healthcare costs, and adversely affect patient recovery. Identifying effective wound management techniques to minimize these complications is crucial. The US Centers for Disease Control and Prevention (CDC) defines SSIs as infections of the incision, organ, or space infections that occur after surgery in that part of the body. They are classified as superficial (involving the skin and subcutaneous tissue), deep (involving tissues such as the fascia and muscle), and organ/space SSIs. The CDC has defined objective clinical criteria for superficial, deep, and organ/space SSIs [2]. Wound complications following emergency laparotomy can significantly impact patients’ quality of life and postoperative recovery. Minimizing wound complications and promoting timely wound healing can enhance patient comfort, satisfaction, and overall quality of life during recovery. Various interventions have been examined over time to find ways to reduce the burden of SSIs [3].

Negative-pressure wound therapy (NPWT) is a novel, practical, and relatively recent tool employed by surgeons in the care of wounds (including incisions) with the intent to control surgical-site occurrences, including SSIs, based on a landmark experimental study by Morykwas et al. in a porcine model in 1997 [4]. NPWT assists in wound healing by producing micro-deformation and hypoxia at the cellular level, favoring a milieu conducive to the activation of cytokine pathways promoting cellular proliferation and angiogenesis, eliminating wound exudates, and favoring a microclimate in the wound that hinders bacterial overgrowth. NPWT might also benefit by preventing unnecessary dressing changes and reducing exposure to the environment, reducing the chances of external contamination as a source of infection [5]. Many studies have evaluated the effect of prophylactic NPWT on open and closed incisional wounds in several surgical specialties, showing a decrease in the incidence of postoperative SSIs. However, the application of NPWT to high-risk delayed primary closed (DPC) abdominal incisions following emergency laparotomy still needs further studies. Identifying effective strategies for wound management can formulate clinical guidelines, standardize protocols, and improve surgical outcomes across healthcare settings [6]. Our study hypothesizes that the application of NPWT, followed by DPC, would reduce the incidence of postoperative SSIs in cases of intestinal perforation.

## Materials and methods

A prospective, observational, cohort study was conducted among 150 patients who underwent emergency exploratory laparotomy due to intestinal perforation at the general surgery department of a tertiary care hospital between July 2022 and December 2023. Institutional Review Board and Ethics Committee of Vardhman Mahavir Medical College & Safdarjung Hospital, New Delhi, India, reviewed and approved this study (approval number: IEC/VMMC/SJH/Thesis/06/2022/CC-1345). Patients with abdominal malignancy, pregnancy, or hemodynamic instability were excluded. Preoperatively, all patients underwent initial resuscitation in the emergency room, nasogastric tube insertion, and urinary catheterization. Intravenous analgesia and antibiotics were administered empirically after the appropriate test dose. Detailed history and examination were performed. Any pre-existing abdominal skin/subcutaneous infections were noted. Appropriate hematological and radiological investigations were conducted. The grade of the patient’s Acute Physiology and Chronic Health Evaluation (APACHE II) and American Society of Anesthesiologists (ASA) were calculated. Patients were then shifted to the emergency operation theater for exploratory laparotomy. Intraoperatively, the extent of peritonitis was determined and categorized according to the CDC classification, originally devised to describe the severity of contamination [2].

Postoperatively, NPWT dressing was applied to the patient’s midline laparotomy wound on postoperative day (POD) two. The NPWT device consists of a vacuum pump, a canister, connecting tubes, sterile porous foam, and semi-occlusive dressing. The device covers the abdominal wound, acting as a temporary dressing. Negative pressure was set at 75-125 mmHg with suction, which was continued for four days. After this, NPWT was removed for wound assessment, and culture and sensitivity of tissue/pus were sent. NPWT was repeated in the same manner until the wound was clean and granulated. The number of NPWT dressing changes required and evidence of any NPWT-related complication were documented. The wound was closed with vertical mattress sutures under local anesthesia, i.e., DPC. After the wound was closed, the occurrence of SSI was accessed every day as per the Southampton wound grading system. The patient was discharged once they were vitally stable, mobile, accepting orally, passing flatus/stools (or having a functional ostomy), passing urine, and the post-DPC wound was free of complications. The patient was followed until POD 30. The patient was assessed for any SSIs after discharge on outpatient department visits. If any evidence of SSI was found, it was managed with dressings and appropriate antibiotics as per the culture and sensitivity.

The primary objective was to evaluate the incidence of SSIs in abdominal wounds after applying NPWT followed by DPC. The secondary objectives were to assess the duration of DPC, the Incidence of fascial dehiscence, the number of NPWT dressing changes, the incidence of NPWT-related complications, and the length of hospital stay. Descriptive statistics were analyzed using the latest SPSS software (IBM Corp., Armonk, NY, USA). Continuous variables were presented as mean ± SD. Categorical variables were expressed as frequencies and percentages. The comparison of normally distributed continuous variables between the groups (according to the incidence of SSI) was performed using Student’s t-test. Non-normal distribution continuous variables were compared using the Mann-Whitney U test. Nominal categorical data between the groups were compared using the chi-square or Fisher’s exact test as appropriate. A p-value less than 0.05 was considered for all statistical tests to indicate a significant difference.

## Results

The analysis of CDC scores across different age groups revealed that most patients’ wounds belonged to CDC 2, followed by CDC 3 and CDC 4. The mean age in classes CDC categories 2, 3, and 4 were 31.789, 28.733, and 42.676 years, respectively. Statistical tests confirmed significant differences in mean scores among age groups (F = 12.604, p < 0.0001) (Table 1). The analysis of CDC scores according to gender revealed no significant association between the two variables (p = 0.15). Among females, the distribution of CDC scores showed 33.3% for CDC 2, 43.3% for CDC 3, and 23.3% for CDC 4. Conversely, among males, the distribution is 50.8% for CDC 2, 26.7% for CDC 3, and 22.5% for CDC 4 (Table 1). The most common cause of perforation was enteric fever (n = 42, 28%), followed by tuberculosis (TB) (n = 36, 24%) (Table 2). Statistical analysis revealed a significant association between the cause of perforation and CDC distribution (χ² = 40.097, p = 0.005). Most patients had no known comorbidities (n = 80, 53.3%). Overall, 16% of patients (n = 24) were both alcoholics and smokers (Table 3).

**Table 1:** Distribution of type of wound (CDC categories) according to age and gender. *: p-value <0.05 is taken as significant. CDC = Centers for Disease Control and Prevention

CDC	2	3	4	F-value	P-value
Mean age	31.789	28.733	42.676	12.604	<0.0001*
Male	61 (50.8%)	32 (26.7%)	27 (22.5%)	3.782	0.151
Female	10 (33.3%)	13 (43.3%)	7 (23.3%)		

**Table 2:** Distribution of CDC categories according to the cause of perforation. *: p-value <0.05 is taken as significant. CDC = Centers for Disease Control and Prevention; TB = tuberculosis

Cause of perforation	CDC 2	CDC 3	CDC 4	Total	P-value
None	1 (100.0%)	0	0	1	0.005*
Appendicitis	4 (57.1%)	1 (14.3%)	2 (28.6%)	7	
Appendicolith	3 (42.9%)	3 (42.9%)	1 (14.3%)	7	
Diverticulitis	1 (33.3%)	2 (66.6%)	0	3	
Enteric perforation	16 (38.1%)	17 (40.5%)	9 (21.4%)	42	
Liver abscess with typhlitis	0	1 (14.3%)	6 (85.7%)	7	
Penetrating injury	3 (100%)	0	0	3	
Peptic ulcer	18 (60.0%)	8 (26.7%)	4 (13.3%)	30	
TB	13 (36.1%)	12 (33.3%)	11 (30.6%)	36	
Traumatic	12 (85.7%)	1 (7.1%)	1 (7.1%)	14	
Total	71	45	34	150	

**Table 3:** Distribution of CDC categories according to comorbidities. *: p-value <0.05 is taken as significant. CDC = Centers for Disease Control and Prevention; DM = diabetes mellitus; HTN = hypertension; TB = tuberculosis

Comorbidity	CDC 2	CDC 3	CDC 4	Total	P-value
No comorbidity	37 (46.3%)	31 (38.8%)	12 (15.0%)	80	0.028*
DM	5 (38.5%)	1 (7.7%)	7 (53.8%)	13	
HTN	3 (60.0%)	0	2 (40.0%)	5	
Obesity	7 (77.8%)	2 (22.2%)	0	9	
Obesity/DM	0	1 (100.0%)	0	1	
Obesity/smoker	1 (100.0%)	0	0	1	
Smoker	1 (100.0%)	0	0	1	
Smoker/alcoholic	12 (50.0%)	6 (25.0%)	6 (25.0%)	24	
Smoker/alcoholic/DM	0	2 (40.0%)	3 (60.0%)	5	
TB	5 (45.5%)	2 (18.2%)	4 (36.4%)	11	
Total	71 (47.3%)	45 (30.0%)	34 (22.7%)	150	

The chi-square test indicated a statistically significant association between the comorbidity and CDC categories (χ² = 33.777, p = 0.028). Patients with perforation due to peptic ulcer showed a higher proportion in CDC category 2 (60.0%) compared to other categories. Those with traumatic perforation predominantly fell under CDC category 2 (85.7%). Patients with perforation due to appendicitis had a higher representation in CDC category 2 (57.1%) and category 4 (28.6%). Enteric perforation cases are distributed across all CDC categories, with slightly higher proportions in categories 2 and 3. Patients with perforation due to TB showed a relatively equal distribution across all CDC categories. Wound swab cultures were sent in all cases (Table 4). In CDC category 2, the highest frequency of bacteria was seen for *Escherichia coli* (*E. coli*) (11 samples)and mixed culture (12 samples). In CDC category 3, the most frequent bacteria were *E. coli* (10 samples) and *Enterococcus* (9 samples). In CDC category 4, the most frequent bacteria were *E. coli* (10 samples) and mixed culture (8 samples). The chi-square value was 28.601, with a p-value of 0.096. There was no statistically significant difference in the distribution of CDC categories across different bacteria types on culture.

**Table 4:** Distribution of CDC categories according to the bacteria extracted from culture. CDC = Centers for Disease Control and Prevention

Bacteria extracted from culture	CDC 2	CDC 3	CDC 4	Total	P-value
Acinetobacter	3 (42.9%)	3 (42.9%)	1 (14.3%)	7	0.096
Bacteroides	8 (47.1%)	6 (35.3%)	3 (17.6%)	17	
Escherichia coli	11 (35.5%)	10 (32.3%)	10 (32.3%)	31	
Enterococcus	5 (29.4%)	9 (52.9%)	3 (17.6%)	17	
Eubacteria	1 (100%)	0	0	1	
Klebsiella	6 (50.0%)	3 (25.0%)	3 (25.0%)	12	
Lactobacillus	1 (14.3%)	2 (28.6%)	4 (57.1%)	7	
Mix culture	12 (54.5%)	2 (9.1%)	8 (36.4%)	22	
No growth	11 (61.1%)	6 (33.3%)	1 (5.6%)	18	
Peptostreptococcus	4 (57.1%)	2 (28.6%)	1 (14.3%)	7	
Streptococcus	9 (81.8%)	2 (18.2%)	0	11	

In total, 14 patients developed burst abdomen in the postoperative period and were excluded from the study. According to the CDC, the DPC duration analysis revealed significant differences among the groups, as indicated by the one-way analysis of variance test (F = 40.562, p < 0.0001). The mean duration of DPC increased with higher CDC categories, with CDC category 4 displaying the most extended mean duration at 10.70 days, followed by category 3 at 9.40 days, and category 2 at 7.34 days (Table 5). These findings suggest that the severity of the clinical diagnosis, as represented by CDC categories, influences the duration of DPC. The analysis of the number of NPWT dressing changes according to the CDC revealed significant differences among the groups, as demonstrated by the chi-square test (χ² = 26.530, p < 0.0001). The number of NPWT dressing changes increased with higher CDC categories, with CDC category 4 exhibiting the highest mean at 2.00, followed by category 3 at 1.84 and category 2 at 1.24 (Table 5). This trend suggests that patients with more severe clinical diagnoses, represented by higher CDC categories, require more NPWT dressing changes.

**Table 5:** Postoperative characteristics in relation to CDC. *: p-value <0.05 is taken as significant. CDC = Centers for Disease Control and Prevention; DPC = delayed primary closure; NPWT = negative-pressure wound therapy; SSI = surgical-site infection

CDC categories	2	3	4	F-value	P-value
n	71	42	23	-	-
Mean duration of DPC	7.34 days	9.40 days	10.70 days	40.567	<0.0001*
Mean number of NPWT dressing changes	1.24	1.84	2.00	26.530	<0.0001*
Mean hospital stay	9.127 days	12.733 days	17.324 days	30.381	<0.0001*
SSI after NPWT + DPC	7 (9.9%)	6 (14.2%)	4 (17.3%)	1.078	0.583
Fascial wound dehiscence	0 (0%)	3 (6.7%)	11 (32.4%)	28.978	<0.0001*
Need for re-admission	7 (9.9%)	5 (11.1%)	9 (26.5%)	5.715	0.057

The remaining postoperative characteristics associated with CDC groups are described in Table 5. The distribution of mean hospital stay according to the CDC demonstrated significant variation among the groups, as indicated by the chi-square test (χ² = 30.381, p < 0.0001). The mean hospital stay increased with higher CDC categories, with CDC category 4 showing the most extended mean stay at 17.324 days, followed by category 3 at 12.733 days, and category 2 at 9.127 days. This trend suggests that patients with more severe clinical diagnoses, represented by higher CDC categories, tend to have more extended hospital stays. The association between SSIs after NPWT followed by DPC and CDC clinical diagnosis categories. Statistical analysis using the chi-square test revealed no significant association between SSI occurrence and CDC categories (χ² = 1.078, p = 0.583).

Statistical analysis using the chi-square test indicated a significant association between fascial wound Dehiscence and CDC categories (χ² = 28.978, p < 0.0001). The occurrence of fascial wound dehiscence varied across different CDC categories (Table 5). CDC category 2 showed no cases of dehiscence. In contrast, as the severity of the CDC category increased, the incidence of dehiscence also increased. Specifically, for CDC category 3, the rate was 6.7%, and for category 4, it increased to 32.4%. These findings suggest a clear trend wherein patients with more severe clinical diagnoses, represented by higher CDC categories, are more likely to experience fascial wound dehiscence. Statistical analysis through the chi-square test indicated no statistically significant association between the need for re-admission and CDC categories (χ² = 5.715, p = 0.057) (Table 5). However, there was a trend of slightly higher re-admission rates with more severe clinical diagnoses.

## Discussion

Our study on NPWT, followed by DPC post-emergency laparotomies, included 150 patients. The subjects in our research predominantly consisted of a younger population, with a mean age of 33.34 years, similar to a survey by Lozano-Balderas et al. [7] (mean age = 32 years). This can be explained by our study’s most common cause of laparotomy of infective causes, which is prevalent in younger populations. On the other hand, studies by Kugler et al. [8] (mean age = 47 years), Frazee et al. [9] (mean age = 54 years), Liu et al. [10] (mean age = 61 years), and Ota et al. [11] (mean age = 68 years) consisted predominantly of elderly populations. This can be explained by the fact that the most common cause of laparotomy in these studies was colorectal malignancy, which is prevalent in the elderly population. CDC has classified surgical wounds into the following four types based on the level of contamination in the operative field: class I: clean, class II: clean contaminated, class III: contaminated, and class IV: dirty. Class II is the operative wound where the gastrointestinal tract is entered under controlled conditions without unusual contamination. Class III refers to open, fresh accidental wounds or conditions with gross uncontrolled spillage from the gastrointestinal tract with acute non-purulent inflammation. Class IV refers to traumatic wounds with devitalized tissue wherein the organism responsible for infection is present in the field preoperatively [2]. In our study, patients were categorized based on the site of perforation as follows: gastric (n = 33), small bowel (n = 85), appendix (n = 14), and large bowel (n = 18), similar to studies by Ota et al. [11] and Sato et al. [12] (Table 6).

**Table 6:** Distribution of the site of perforation reported in previous studies.

Site of perforation	Our study	Ota et al., 2020 [11]	Sato et al., 2022 [12]
Gastric	33	0	5
Small bowel	85	6	10
Appendix	14	14	3
Large bowel	18	36	4
Total	150	56	22

In our study of 150 patients, 90.7% underwent DPCs following NPWT. Among these, only 12.5% experienced subsequent SSIs managed by antibiotics and conventional dressings. Similar results were observed in other studies (Table 7). In a survey by Ota et al. [11], 91% of patients underwent DPC following NPWT and 13.7% developed SSIs. Danno et al. [13] observed that 100% underwent DPC following NPWT and 10.7% developed SSIs. In another study by Liu et al. [10], 100% underwent DPC, followed by NPWT, and 8.6% developed surgical site infection. In another study by Sato et al. [12], an exceptionally high incidence of 31.8% of SSIs was observed. All patients underwent DPC followed by NPWT due to organ/space SSI (27.3%) and incisional SSI (4.5%).

**Table 7:** Postoperative characteristics reported in previous studies.

Studies	Delayed primary closure	Incidence of surgical-site infection	Fascial wound dehiscence	Length of hospital stay
Liu et al., 2021 [10]	100%	8.6%	4.3%	-
Ota et al., 2020 [11]	91%	13.7%	-	-
Sato et al., 2022 [12]	100%	31.8%	-	17 days
Danno et al., 2018 [13]	100%	10.7%	-	22 days
Zaidi et al., 2017 [14]	-	-	2.9%	-
Our study	90.7%	12.5%	9.3%	12 days

Wound dehiscence poses significant concerns after laparotomy in patients undergoing general surgery, particularly in those with various comorbidities and grades of contamination, as they have the potential to occur more frequently. In our study, 14 (9.3%) patients developed fascial wound dehiscence. Out of these, 11 (32%) were from CDC 4, which shows that wound dehiscence is more likely to occur with high contamination rates. This is similar to a study by Kugler et al. [8] (Table 7). In contrast, the study by Liu et al. [10] demonstrated a wound dehiscence rate of 4.3%. In another study by Zaidi et al. [14], a 2.9% rate of wound complications was observed. Notably, this study comprised CDC 2 and 3 patients, with no patients belonging to CDC 4. In our study, the mean postoperative length of hospital stay varied among different CDC classes, with CDC 2 at 9.1 days, CDC 3 at 12.7 days, and CDC 4 at 17.3 days. The mean length of hospital stay was 12 days. This closely aligns with the study by Sato et al. [12], which reported a CDC IV length of hospital stay at 17 days. In contrast, other studies by Selvaggi et al. [15] and Liu DS et al. [10] showed a reduced length of hospital stay compared to the control group. However, in the study by Danno et al. [13], the length of hospital stay was 22 days (Table 7). In our study, the number of patients requiring re-admission was 21 (14%), which was not statistically significant. The common reasons for re-admission in our study included electrolyte imbalance (in stoma patients) followed by ileus, which was conservatively managed. SSI was less common as a reason for re-admission. A retrospective case-control study by Liu et al. [10] showed a lower rate of wound-related re-admissions.

Limitations of the study

The study lacked a randomized control group, making it susceptible to selection bias and limiting the ability to establish causality between the intervention and outcomes. The study had a relatively short follow-up duration, which may have precluded assessing long-term outcomes and complications associated with NPWT and DPC. Including patients with different etiologies of intestinal perforation may introduce variability in outcomes, as the underlying pathology and severity of infection could differ. The study’s findings may only apply to some patient populations, as they were conducted in specific settings with our patient demographics and healthcare practices.

## Conclusions

NPWT followed by DPC is a promising approach to managing gastrointestinal perforations. This approach has demonstrated potential in reducing SSIs and wound dehiscence and minimizing the incidence of wound complications. However, additional research is essential to fully understand its effectiveness and benefits across different clinical scenarios. Exploring the role of NPWT in conjunction with DPC in various contexts will provide valuable insights into optimizing patient outcomes in surgical practice.
